# Rationalisation and Validation of an Acrylamide-Free Procedure in Three-Dimensional Histological Imaging

**DOI:** 10.1371/journal.pone.0158628

**Published:** 2016-06-30

**Authors:** Hei Ming Lai, Alan King Lun Liu, Wai-Lung Ng, John DeFelice, Wing Sang Lee, Heng Li, Wen Li, Ho Man Ng, Raymond Chuen-Chung Chang, Bin Lin, Wutian Wu, Steve M. Gentleman

**Affiliations:** 1 School of Biomedical Sciences, Li Ka Shing Faculty of Medicine, The University of Hong Kong, Pokfulam, Hong Kong SAR, China; 2 Neuropathology Unit, Division of Brain Sciences, Department of Medicine, Imperial College London, United Kingdom; 3 Chemistry Research Laboratory, Department of Chemistry, University of Oxford, Oxford, United Kingdom; 4 Electron Microscope Unit, The University of Hong Kong, Pokfulam, Hong Kong SAR, China; 5 State Key Laboratory of Brain and Cognitive Sciences, Li Ka Shing Faculty of Medicine, The University of Hong Kong, Pokfulam, Hong Kong SAR, China; 6 Research Centre of Heart, Brain, Hormone and Healthy Aging, Li Ka Shing Faculty of Medicine, The University of Hong Kong, Pokfulam, Hong Kong SAR, China; 7 School of Optometry, The Hong Kong Polytechnic University, Hung Hom, Hong Kong SAR, China; 8 Joint Laboratory of Jinan University and The University of Hong Kong, GHM Institute of CNS Regeneration, Jinan University, Guangzhou, China; The University of Akron, UNITED STATES

## Abstract

Three-dimensional visualization of intact tissues is now being achieved by turning tissues transparent. CLARITY is a unique tissue clearing technique, which features the use of detergents to remove lipids from fixed tissues to achieve optical transparency. To preserve tissue integrity, an acrylamide-based hydrogel has been proposed to embed the tissue. In this study, we examined the rationale behind the use of acrylamide in CLARITY, and presented evidence to suggest that the omission of acrylamide-hydrogel embedding in CLARITY does not alter the preservation of tissue morphology and molecular information in fixed tissues. We therefore propose a novel and simplified workflow for formaldehyde-fixed tissue clearing, which will facilitate the laboratory implementation of this technique. Furthermore, we have investigated the basic tissue clearing process in detail and have highlighted some areas for targeted improvement of technologies essential for the emerging subject of three-dimensional histology.

## Introduction

The opacity of tissues arises as a result of light scattering at the boundaries of the heterogeneous intermix of hydrophilic and hydrophobic components with different refractive indices within the tissue. [*1,2*] A technique called CLARITY [*1*] has been developed by delipidating tissues with the detergent sodium dodecyl sulphate (SDS), which removes the major hydrophobic components to achieve tissue clarity for deep microscopic imaging. Proposed by its inventors to preserve tissue integrity, CLARITY includes a protective hydrogel-embedding step prior to delipidation. In this study we examined the theoretical aspects behind the practically complex hydrogel-embedding step, and demonstrated that a formaldehyde-crosslinked tissue serves as an effective protective physical framework for detailed three-dimensional histological evaluation, leading us to propose the omission of acrylamide embedding in CLARITY. The result is a vastly simplified workflow with potentially added benefits, which includes the avoidance of toxic acrylamide, increased antibody penetration, reduced tissue expansion, and compatibility with other tissue processing techniques [*3*].

The original CLARITY technique included the hydrogel-embedding step based on two hypothetical reasons [*1*]: firstly, delipidation removes lipid bilayers essential for cellular integrity; secondly, acrylamide might be used to crosslink formaldehyde-modified amines (called formaldimines) on proteins via a nucleophilic addition reaction. The attached acrylamide will then be polymerized, forming a physical framework to prevent excessive protein loss from tissues during SDS-mediated delipidation, whereas lipids and other biomolecules lacking the amine groups can be washed off. Intuitively, this is an attractive hypothesis but there is little experimental evidence to support it and there is no available comparisons on tissue morphologies between acrylamide-embedded and non-embedded samples. We noticed that *tissue* integrity is largely maintained by proteins, whereas *cellular* integrity is largely dependent on the plasma membrane. We also questioned the proposed amide-formaldimine nucleophilic addition reaction because the acrylamide nitrogen is weakly nucleophilic, and is debatable whether such a reaction functions to a significant extent *in situ*. Therefore, we aimed to investigate the role of an acrylamide-based hydrogel in tissue clearing by SDS-mediated delipidation (hereafter referred to as clearing), and subsequently we propose the replacement of the complex hydrogel-embedding step by standard passive formaldehyde-fixation.

## Materials and Methods

### Chemicals and reagents

Chemicals and reagents: Acrylamide (VWR BDH Prolabo, Electran® 40% w/v solution, catalogue number 443545P) and bisacrylamide (national diagnostics, 2% w/v solution, Protein and Sequencing Electrophoresis grade, catalogue number EC-820) were checked for any discoloration to ensure purity and was used as received. VA-044 Thermal initiator (Wako chemicals) was used as received.

### Antibodies

The antibodies used in this study are listed in **Table 1.**

**Table 1:** Antibodies used in this study.

Antibody	Company, Catalogue number (Lot number)
Anti-MAP2, rabbit polyclonal	Millipore AB5622 (2202428)
Anti-Neurofilament, mouse monoclonal	Dako, M0762 (1495837)
Anti-β-Tubulin III, mouse monoclonal	Sigma, T8660 (082M4845)
Anti-Tyrosine Hydroxylase, rabbit polyclonal	Millipore, AB152 (2458991)
Donkey anti-Goat IgG, AlexaFluor®488 conjugate	Invitrogen, A11055 (1463163)
Donkey anti-Mouse IgG, AlexaFluor®488 conjugate	Invitrogen, A21202 (1644644)
Donkey anti-Rabbit IgG, AlexaFluor®568 conjugate	Invitrogen, A10042 (1020757)
Donkey anti-Mouse IgG, AlexaFluor®568	Invitrogen, A10037 (1495837)
Goat anti-Rabbit IgG, AlexaFluor®594	Invitrogen, A11012 (1515530)

### Animals

All animals used in this study were approved and handled in accordance with the guidelines provided by the Committee on the Use of Live Animals in Teaching and Research (CULATR) in the Laboratory Animal Unit, HKU with approval (CULATR reference number: 3699–15). All mouse brain tissues were harvested immediately after euthanasia using intraperitoneal sodium pentobarbital (150mg/kg), flushed clean with normal saline, perfusion fixed with 4% PBS-buffered PFA for 10 minutes, and immersed in the same fixation solution for at least 2 days (to up to 9 months, as individually specified for each sample) at 4°C before proceeding to the CLARITY procedure.

### Human samples

Human brain tissues used in this study was provided by the Parkinson’s UK Tissue Bank at Imperial College London, which have all been fixed in 10% buffered formalin for at least 3 weeks before proceeding to CLARITY.

### Model protein reaction for CLARITY

Lysozyme (10 mg) was dissolved in 1x PBS buffer (500 μL) containing formaldehyde (4%). The mixture was shaken at room temperature for 15 minutes. 38μL of acrylamide-bisacrylamide (37.5:1) solution 30% was added to the above mixture to a final acrylamide concentration of 4%. For control experiment, 38μL of 1x PBS was added instead. The reaction mixture was incubated at 4°C for 1 day. The reaction progress was monitored by LC-MS.

### Protein Mass Spectrometry

Liquid chromatography-mass spectrometry (LC-MS) was performed on a Micromass LCT (ESI-TOF-MS) coupled to a Waters Alliance 2790 HPLC using a Phenomenex Jupiter C4 column (250 x 4.6 mm x 5μm). Water:acetonitrile, 95:5 (solvent A) and acetonitrile (solvent B), each containing 0.1% formic acid, were used as the mobile phase at a flow rate of 1.0 mL min^-1^. The gradient was programmed as follows: 95% A (5 min isocratic) to 100% B after 15 min then isocratic for 5 min. The electrospray source of LCT was operated with a capillary voltage of 3.2 kV and a cone voltage of 25 V. Nitrogen was used as the nebulizer and desolvation gas at a total flow of 600 L hr^-1^. Spectra were calibrated using a calibration curve constructed from a minimum of 17 matched peaks from the multiply charged ion series of equine myoglobin, which was also obtained at a cone voltage of 25V. Total mass spectra were reconstructed from the ion series using the MaxEnt algorithm preinstalled on MassLynx software (v. 4.0 from Waters) according to manufacturerʼs instructions.

### Passive CLARITY

The available protocol was followed except for several points: (1) omission of acrylamide and bisacrylamide in the hydrogel monomer solution for our experiments, which were replaced with water; (2) use of 8% SDS in 1x PBS instead of Sodium borate clearing buffer (SBC) in some of the experiments specified above; (3) for refractive index-matching, Histodenz-RIMS was used for mouse brain, and 47% 2,2’-Thidiethanol in 10mM phosphate buffer was used for human brain tissues.

### Electrophoretic tissue clearing (ETC)

A ETC setup was self-constructed using a lunchbox, platinum wires, DC power supply, a fish tank water pump, and a water bath (**S7 Fig**). The temperature, pH of the clearing buffer, running voltage and current, were all monitored hourly and kept stable by manual adjustments.

### Conventional histology

Clarified tissues (acrylamide-embedded or non-embedded) were dehydrated in an automatic processor and embedded in paraffin, and sectioned at 20-μm thickness using a microtome. Hematoxylin & Eosin staining was performed according to the Standard Operating Procedures provided by the Parkinson’s UK Tissue Bank (Procedure number MSP-S-033 and MSP-S-034, respectively). Briefly, tissues were stained in Meyer’s hematoxylin for 5 min, washed in hard tap water for 5 min, stained in eosin for 5 min, washed very briefly in water, and dehydrated through series of ethanol and xylene before mounting in Paramount.

### Immunohistochemistry

All antibodies were used at 1:100 dilutions in PBST with 0.01% w/v NaN_3_. Samples were stained at 37°C for 2 days. Sequential staining was employed for double immunohistochemistry, DAPI was added to a final concentration of 1 ng/ml when desired from a stock of 1 mg/ml in DMSO.

### Confocal Imaging

Imaging was performed using a Leica SP5 Confocal Microscope (objectives used: HCX PL APO CS 10.0x (NA 0.40) DRY UV, HCX PL APO CS 20.0x (NA 0.70) DRY UV, and HCX PL APO 40.0x (NA 0.85)), and a Carl Zeiss LSM 780 Confocal Microscope (objectives used: EC Plan-Neofluar 5x (NA 0.16) Ph1 M27, Plan-Apochromat 10x (NA 0.45) Ph1 M27, Plan-Apochromat 20x (NA 0.8) Ph2 M27, Plan-Apochromat 40x (NA 1.4) Oil DIC M27, Plan-Apochromat 100x (NA 1.40) Oil Ph3 M27)

### Confocal Image analysis

Confocal image maximum intensity projections and Z-depth color-coding were performed using Fiji and Zen Black software, 3D renderings were performed using Imaris 7.2.3. Scanning electron micrographs were cropped and contrast-adjusted using Adobe After Effects CS6 software.

### Scanning electron microscopy

Samples were processed according to the standard procedures provided by the Electron Microscopy Unit of the University of Hong Kong (http://www.emunit.hku.hk/documents/SamplePreparationTechnique.pdf, page 27, starting from step 6). Briefly, samples were dehydrated in a series of ethanols, dried in a BAL-TEC CPD 030 Critical Point Dryer using liquid carbon dioxide as transitional fluid, and gold-coated using a BAL-TEC SCD 005 Sputter Coater/Carbon Coater. Images were acquired using a Hitachi S-4800 FEG scanning electron microscope and a LEO 1530 FEG scanning electron microscope.

### Protein assay and SDS-PAGE

The Bradford assay (ThermoScientific #1856209) was employed for rapid protein measurement in sample aliquots, absorbance at 595 nm was measured using a UV spectrophotometer (Perkin Elmer Victor^3^ 1420 Multilabel Counter) in a 96-well plate. SDS-PAGE was performed using 6% stacking gel and 15% separating gel, ran at 120 V for approximately 2.5 hours, and stained with 1% Coomassie Brilliant Blue 250R in destaining buffer overnight. The stained gels were destained in destaining buffer (1:4:5 acetic acid:methanol:water) until no background blue hue was observed and photographed using a digital camera.

### Protease digestion

1% acrylamide-embedded, cleared mouse brain slices of 2 mm thick were digested separately with Proteinase K, 0.1 mg/ml in PBST; or *Clostridium histolyticum* Collagenase (Sigma C0130-100MG, lot. no. SLBJ7761V), 0.1 mg/ml in PBS with 0.018 mM CaCl_2_ at 37°C overnight.

## Results

Based on our theoretical considerations, we started off observing SDS-mediated delipidation of hydrogel-embedded and non-embedded tissues. We found that non-embedded tissues can be cleared without the significant tissue expansion seen in hydrogel-embedded tissues (**S1A Fig**). Moreover, we observed that the time taken to adequately delipidate a tissue block depended largely on the conditions of formaldehyde fixation instead of the concentration of acrylamide used for embedding (data not shown), and the usage of ETC did not alter the time course of tissue clearing. Importantly, the preservation of structural integrity of tissues during SDS-mediated delipidation depended more on the formaldehyde fixation conditions, but little on whether acrylamide embedding has been performed (**S1B Fig,**. see also discussion below). We next systematically varied the acrylamide/bisacrylamide/formaldehyde combinations used for embedding and quantified the amount of protein loss from tissues using the Bradford assay and SDS-PAGE analysis. Interestingly, a poor correlation between the embedding formulation used and the amount of protein loss was found (**S2 Fig**).

**Fig 1:**
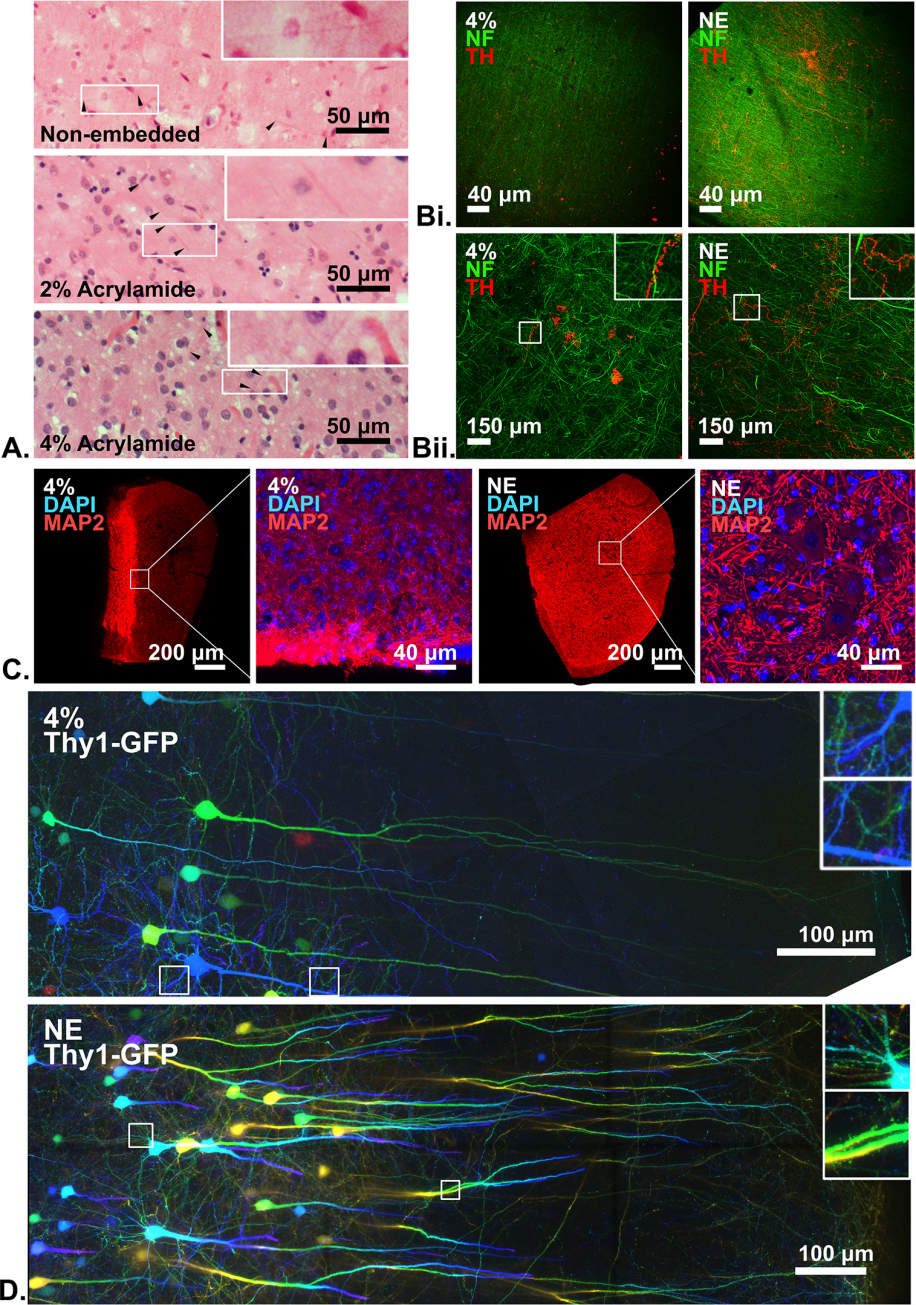
Histological comparisons between non-embedded samples and acrylamide-embedded samples cleared in SDS. All imaging parameters have been controlled for each comparisons. Insets in selected figures shows enlarged views from their respective images. 4%: 4%-acrylamide embedded; NE: non-embedded samples. Scale bars as labelled within the figures. (**A**.) Haematoxylin & Eosin-stained, 20-μm paraffin-embedded sections of cleared human brain tissues that have been embedded in various hydrogel formulations as labelled. Fine neural fibers were preserved and visible in all cases (arrowheads), and some eosinophilic cell soma were also visible. (**B-D.)** Confocal microscopy. (**Bi**.) Comparison using human occipital lobe tissue, stained for neurofilament (NF, green) and tyrosine hydroxylase (TH, red) under 10x magnification. Maximum intensity projection, Z-depth 490 μm. Note that the TH-positive fibers/signals were more dispersed in the 4%-acrylamide embedded sample but remained traceable in the non-embedded sample, probably because the latter was less swollen. (**Bii.**) The same samples under 40x magnification. Maximum intensity projection, Z-depth 100 μm. A higher resolution image of the non-embedded sample can be found in **S3A Fig**. (**C**.) Comparison using mouse spinal cord stained for microtubule-associated protein 2 (MAP2, red) and stained with DAPI (blue). A mouse spinal cord transverse segments was split into two halves for comparison. In the 4% acrylamide-embedded sample the antibody penetration was inadequate such that anterior horn cells were not as clear as seen in the non-embedded sample. For overview images, Z-depths for 4% acrylamide-embedded sample and non-embedded were 170.94 μm and 59.94 μm, respectively. For enlarged views, Z-depths are 18.54 μm. See also **S3B Fig.** for comparisons of other antigens. (**D.)** Comparison using Thy1-GFP transgenic mouse brain tissues, where the motor cortex is imaged. Color-coded maximal intensity projection for Z-depth representation. Z-depth 199.80 μm.

We moved on and evaluated the cleared tissue morphologies under the microscope, where all samples have been fixed for at least 2 days at room temperature. Under the same conditions, non-embedded, 2%, and 4% acrylamide-embedded tissues showed little difference in terms of neural tissue morphology in paraffin-embedded sections stained with haematoxylin and eosin (**Fig 1A**) after SDS-delipidation. Immunostaining for neurofilament (a filamentous, insoluble protein), tyrosine hydroxylase, microtubule-associated protein 2, choline acetyltransferase, and βIII-tubulin (globular, soluble proteins) showed that the non-embedded samples are not inferior to the embedded ones in terms of staining intensities and qualities (**Fig 1B and 1C, S3 Fig**). Perhaps because the non-embedded samples are less swollen, TH-positive fibers are clearer and appear to be less fragmented. In all case, the antibody penetration was limited due to rapid consumption of antibodies by the dense antigens in tissues. This was made worse in 4% acrylamide-embedded samples because the hydrogel imposed further restriction to diffusion. Comparison using Thy1-GFP line M transgenic mouse brain slices also suggests that acrylamide is unnecessary for preserving endogenous fluorescence (**Fig 1D)**.

**Fig 2:**
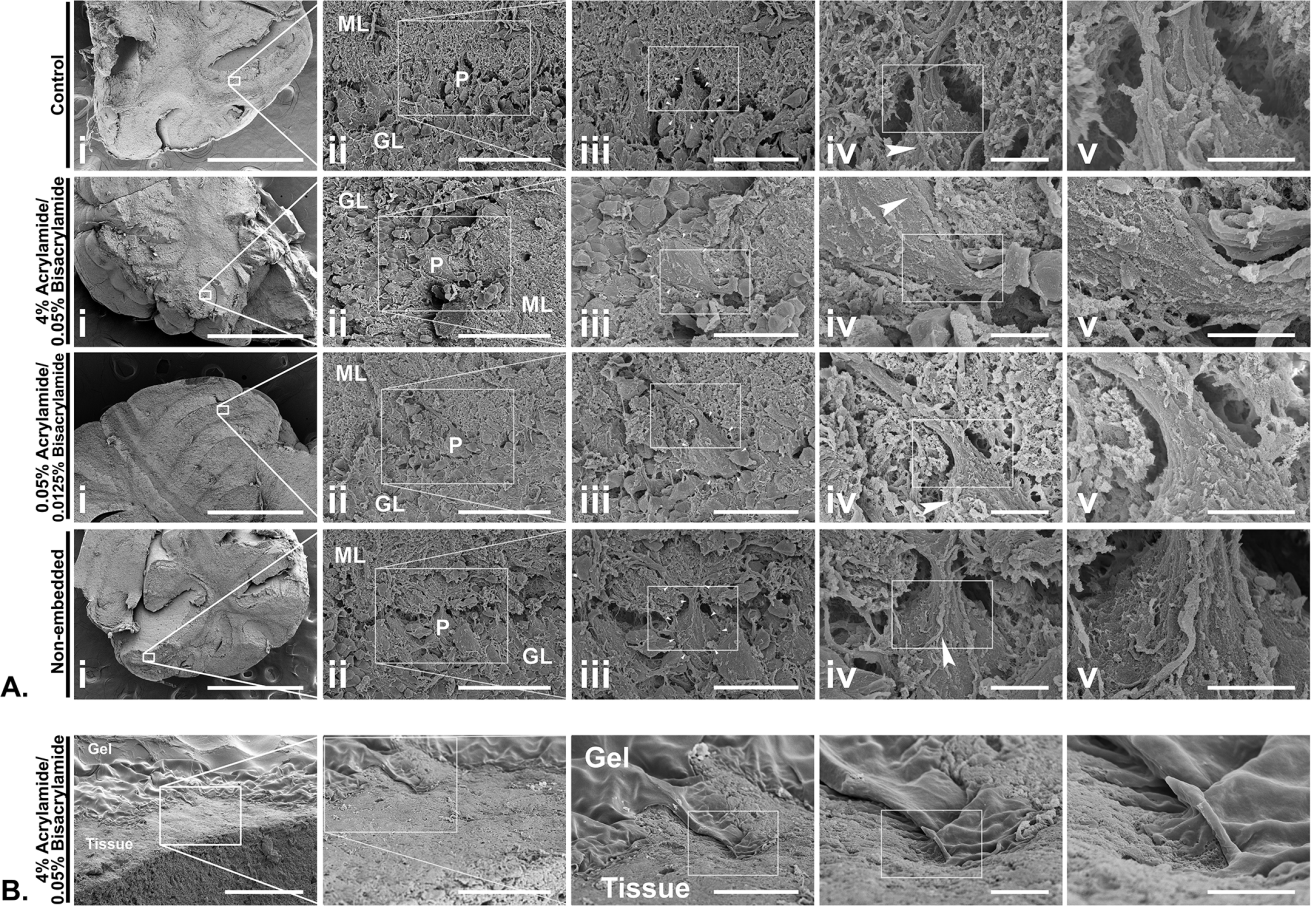
Scanning electron micrographs (SEMs) of clarified, fixed mouse cerebellum slices. Slices embedded in different formulations of acrylamide/bisacrylamide, non-embedded slices, and a non-clarified, non-embedded control was processed and incubated simultaneously in PBST. All four slices were from the same mouse cerebellum (**A.)** Series of SEMs of each sample, in which fortuitous cut surfaces demonstrate Purkinje cells between the molecular and granular layers (labelled as P, ML, and GL respectively in column **ii** of each series). The thick, prominent dendrites of the featured Purkinje cell are visible in all samples, along with climbing fibers (arrow heads), and mossy fibers in the granular layer. The featured Purkinje cells has been outlined in column **iii** of each series. Note that the superficial hydrogel of 4% acrylamide-embedded sample has been sliced off after embedding to reveal the currently imaged tissue surface. Scale bars of each columns from left to right: 1 mm, 40 μm, 20 μm, 5 μm, 3 μm. **(B.)** The same 4% acrylamide-embedded sample demonstrating the tissue-hydrogel interface as labelled, revealed by slicing off the superficial hydrogel in the embedded sample. Note that the hydrogel is somewhat retracted from the tissue due to the dehydration process for scanning electron microscopy, which makes it appears dense. The original porous surface morphology, as well as additional morphologies of the hydrogel can be found in **S4 Fig.** Scale bars from left to right: 100 μm, 40 μm, 20 μm, 5 μm, 3 μm.

At the ultrastructural level, scanning electron micrographs (SEMs) showed no difference in the surface morphology between embedded and non-embedded samples that were fixed, cleared, and processed under the same conditions. Neurons and neurites are clearly visible in all cases, with good preservation of tissue ultrastructure and cellular morphology (**Fig 2A, S4A and S4B Fig**). The polyacrylamide gel itself is seen as a porous matrix on the surface of the 4% acrylamide-embedded sample (**Fig 2B, S4C Fig**).

**Fig 3:**
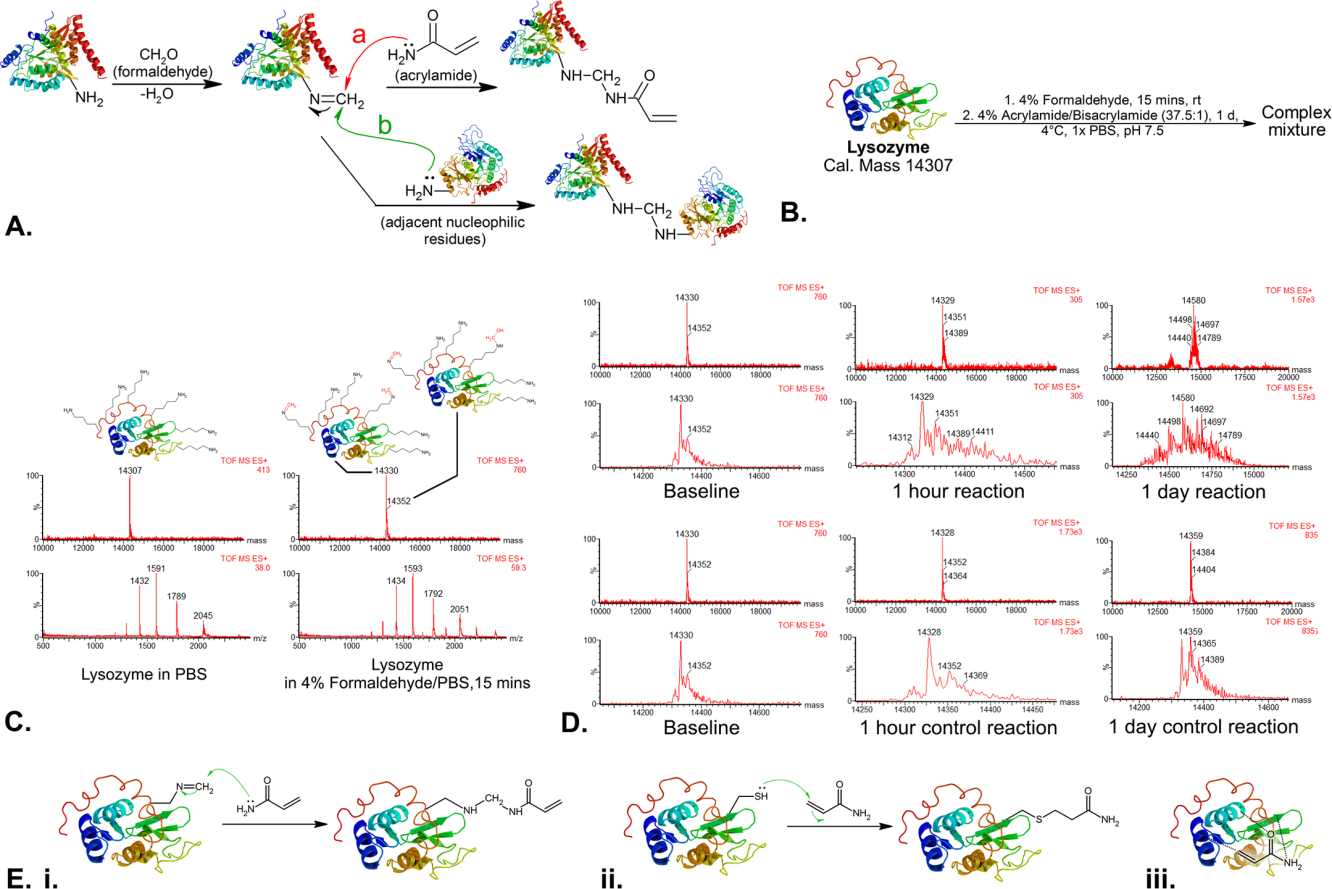
The chemistry background of the CLARITY tissue hydrogel. **(A.)** The proposed CLARITY hydrogel-tissue hybridization reaction (path **a**), where acrylamide’s nitrogen atom is hypothesized to attack the formaldimine carbon formed when tissue proteins were allowed to react with formaldehyde; whilst it has been well-established that protein amines can attack the formaldimine carbons (path **b**), leading to protein cross-linkage which is the basis of conventional fixation. (**B.)** Our model reaction using lysozyme as the model protein to simulate the CLARITY protein-hydrogel hybridization for protein mass spectrometry analysis. (**C**.) Baseline mass spectrometries of lysozyme in PBS (left) and 15 minutes after lysozyme has been allowed to react with 4% formaldehyde in PBS (step 1 of the model reaction in **B**), demonstrating rapid addition of methylene (=CH_2_, +12 *m/z*) and methylol (—CH_2_OH, +30 *m/z*) groups as depicted; the slight ±1 to 2 *m/z* peak shifts from theoretical calculations are likely due to changes in the protein protonation status. (**D**.) Step 2 of model reaction in **B** monitored by protein mass spectrometry, in which formaldehyde-modified lysozyme is allowed to react with 4% acrylamide:bisacrylamide (in 37.5:1 ratio) (upper panel) or 1x PBS (control, lower panel). The reaction mixture was analyzed using LC-MS 1 hour and 24 hours after the reaction started at 4°C. (**E.)** Possible reactions between a formaldehyde-modified protein with acrylamide. (**i.)** Nucleophilic addition of the amide nitrogen to the formaldimine group, as proposed in CLARITY. (**ii.)** Nucleophilic addition of thiol groups from cysteine residues in proteins to the Michael-receptive double bond of acrylamide, rendering it unable to crosslink to the polyacrylamide meshwork by polymerisation. (**iii.)** The formation of simple adducts, which potentially explains the heterogeneity and noise seen in the spectrometry data.

With the above comparisons, we deduced that acrylamide plays a relatively insignificant role *in situ* in CLARITY. A review of current literatures [*4–8*] provided an inconclusive argument as to whether the proposed acrylamide nitrogen can indeed function as a nucleophile to attack the formaldimines as proposed in CLARITY (**Fig 3A,** path **a**), possibly due to the different reaction conditions employed. Therefore, we designed a model reaction (**Fig 3B**) with protein mass spectrometry to examine whether acrylamide can significantly modify a protein in the presence of formaldehyde and under the conditions employed in CLARITY. As seen in **Fig 3C and 3D** (and **S5 Fig)**, further formaldehyde modification of formaldehyde-fixed lysozyme occurred quickly within an hour, whilst the effect of acrylamide takes 24 hours, manifested as a global right-shift of the *m/z* peaks heterogeneously and the generation of significant noise in the peak patterns, making it difficult to identify the precise modifications that actually occurred. It appears that acrylamide does play a role in modifying lysozyme under CLARITY conditions, but whether this occurred in the desired manner as in **Fig 3A** is unknown (see discussion below and **Fig 3E**).

## Discussion

Our results suggest that given sufficient fixation, a hydrogel would be unnecessary to protect tissues during aggressive delipidation. Although cells burst when their plasma membranes are disrupted, *cellular* integrity differs from *tissue* integrity. While the former might be largely maintained by plasma membranes, tissues are mechanically robust and their integrity is maintained by an array of intracellular cytoskeletons, cooperative transmembrane adhesion junctions, and extracellular connective components [9]. Moreover, the fixation process of the cytoplasm as a gel with extremely high protein densities [10] provides further stabilization by crosslinking proteins in three dimensions, forming a natural barrier against protein leakage as well as antibody penetration [11]. In support of the hypothesis that proteins, but not lipids, are the key to tissue integrity, the non-specific protease proteinase K can digest and dissolute 1% acrylamide-embedded, SDS-delipidated brain slices completely into a homogeneous solution in 3 hours at 37°C, while with the more specific protease *Clostridium histolyticum* collagenase, a friable brain slice with discernible anatomy remained even with overnight digestion **(S6 Fig).** Finally, SDS denatures proteins [12] and thus their adhesive interactions, which might account for the necessity of tissue protection either by fixation or hydrogel embedding.

Although our protein mass spectrometry data suggests that acrylamide does interact with proteins in some way in our model reaction, the exact reaction products remained obscure, as the heterogeneous peak changes can reflect either (1) nucleophilic addition of acrylamide to formaldimines (**Fig 3E, reaction i)**, (2) nucleophilic addition of cysteines, lysine or histidine to the Michael-receptive double bond of acrylamide (which would render it unavailable to crosslink to the hydrogel meshwork, **Fig 3E, reaction ii**), or (3) formation of simple adducts (**Fig 3E, reaction iii)**. Further, protein crosslinking rigorously occurs during tissue fixation [6–8], consuming the reactive formaldimines essential for the addition of acrylamide (path **b** in **Fig 3A**) in the desired manner. Such protein cross-linkage is absent in an *in vitro* model reaction and/or undetectable with current protein mass spectrometry technologies due to the large mass of the crosslinked proteins. The relative significance of these ‘side reactions’ is uncertain, therefore whether acrylamide can react with formaldehyde-modified proteins as proposed in CLARITY *in situ* is uncertain. Although the CLARITY hydrogel monomer solution contains 4% paraformaldehyde (PFA) that might generate more formaldimines for acrylamide to react with, omission of the 4% PFA suggested by an alternative version of CLARITY gave almost equal results in terms of tissue protein retention and immunolabeling quality [13,14]. This indicates that either a conventional fixation would have consumed and saturated most formaldimines and other residues reactive to formaldehyde, or the proposed acrylamide cross-linkage reaction does not occur to a significant extent *in situ*. In keeping with this, studies on the amount of protein lost into the clearing buffer by us (**S3 Fig**) and others [1,13,14] found only a mild increase in protein lost from non-embedded samples compared with those embedded in acrylamide, most likely attributable to the diffusion constraints of the hydrogel against protein leakage rather than proteins stably crosslinked to a physical meshwork.

Interestingly, our SEMs and deductions above suggest that we could expect minimal increase in immunolabeling speed even when the hydrogel is omitted, as this merely eliminates the flow restriction of antibodies created by the porous hydrogel, not the diffusion limitations caused by the inherent high density of tissues. This is supported by Yang *et al*’s [13] in which they significantly improved the porosity of the hydrogel matrix by altering the hydrogel formulation. The mean pore sizes presented by Yang *et al* before and after their improvement, were 171- and 310-times larger than the hydrodynamic diameter of an antibody in PBS (about 11 nm) [15–17], respectively. In both cases, the pore sizes were significantly large and thus unlikely to be the major limitation to diffusion of antibodies compared with the dense formaldehyde-crosslinked proteinaceous tissue. Therefore, efficient methods of molecular phenotyping are needed to maximize the potential of CLARITY.

Finally, we did not observe a significant improvement in the speed of clearing with decreased concentrations or even omission of acrylamide hydrogel, in contrast with the observations of other groups [1,16]. We believe this is due to four potential confounding factors: (1) the intrinsically dense tissue matrix could represent the prime limitation of diffusion as outlined above, (2) the speed of clearing depends largely on the region of the brain. For example, a 1 cm-thick human hippocampus fixed for 3 weeks would clear consistently in 5 days, whereas a human occipital cortex of similar size, thickness, and fixation timing required >40 days to clear. (3) As mentioned above, the speed of clearing is largely dictated by the fixation timing and conditions, meanwhile the concentration of acrylamide used or the usage of electrophoretic clearing apparatus did not alter the time course of tissue clearing (**S1 Fig)**. This is further exemplified by the virtually non-clearable nature of a 1 cm-thick human hippocampus fixed for >50 years. (4) Even though the region of the brain is carefully selected, a striking difference between a cleared 4% acrylamide-embedded tissue and non-embedded tissue is that the former is greatly swollen, perhaps due to the osmotic effect of the hydrogel. The swollen tissue has its refractive index lowered, which matches with the refractive index of the highly concentrated SDS solution [18], causing the tissue to appear clearer than the non-embedded tissue in SDS (in fact the non-embedded tissue is well delipidated even though it remains opaque in SDS, and will eventually turn transparent completely in the refractive index homogenization solution). This also explains why cleared, acrylamide-embedded tissues turn opaque when washed with PBST, due to the lower refractive index of the salt solution compared to SDS [18].

Although we have proved that formaldehyde fixation alone can replace the laborious acrylamide embedding of tissues for subsequent SDS-mediated delipidation and tissue clearing, the determination of whether fixation has been “adequate” is difficult and is out of the scope of this study. In general, a longer fixation time is recommended for subsequent prolonged treatment in SDS. As a rule of thumb, if the diffusion of formaldehyde fixative is not limited, fixing tissues for 3 days at room temperature is sufficient for a mouse’s whole brain for subsequent tissue clearing in 8% SDS, while a whole human post-mortem brain fixed for 3 weeks at room temperature have tolerated up to about 3 months of 8% SDS treatment at 55°C. Further, as mentioned above, since the subsequent immersion of tissues in the refractive index homogenising solutions will aid in making tissues transparent, aggressive delipidation to completion is usually not required. For example, a 2 mm-thick mouse brain slice fixed for 3 days to 1 year can be immersed in 8% SDS for 3 to 7 days at 37°C, after which good transparency can be achieved by immersing the partially delipidated tissue into the iohexol-based refractive index homogenising solution. Such partial delipidation approach saves time and avoids the risk of excessive tissue damage, yet the permeabilisation remains adequate for the penetration of antibodies and chemical dyes.

## Conclusion

**Fig 4:**
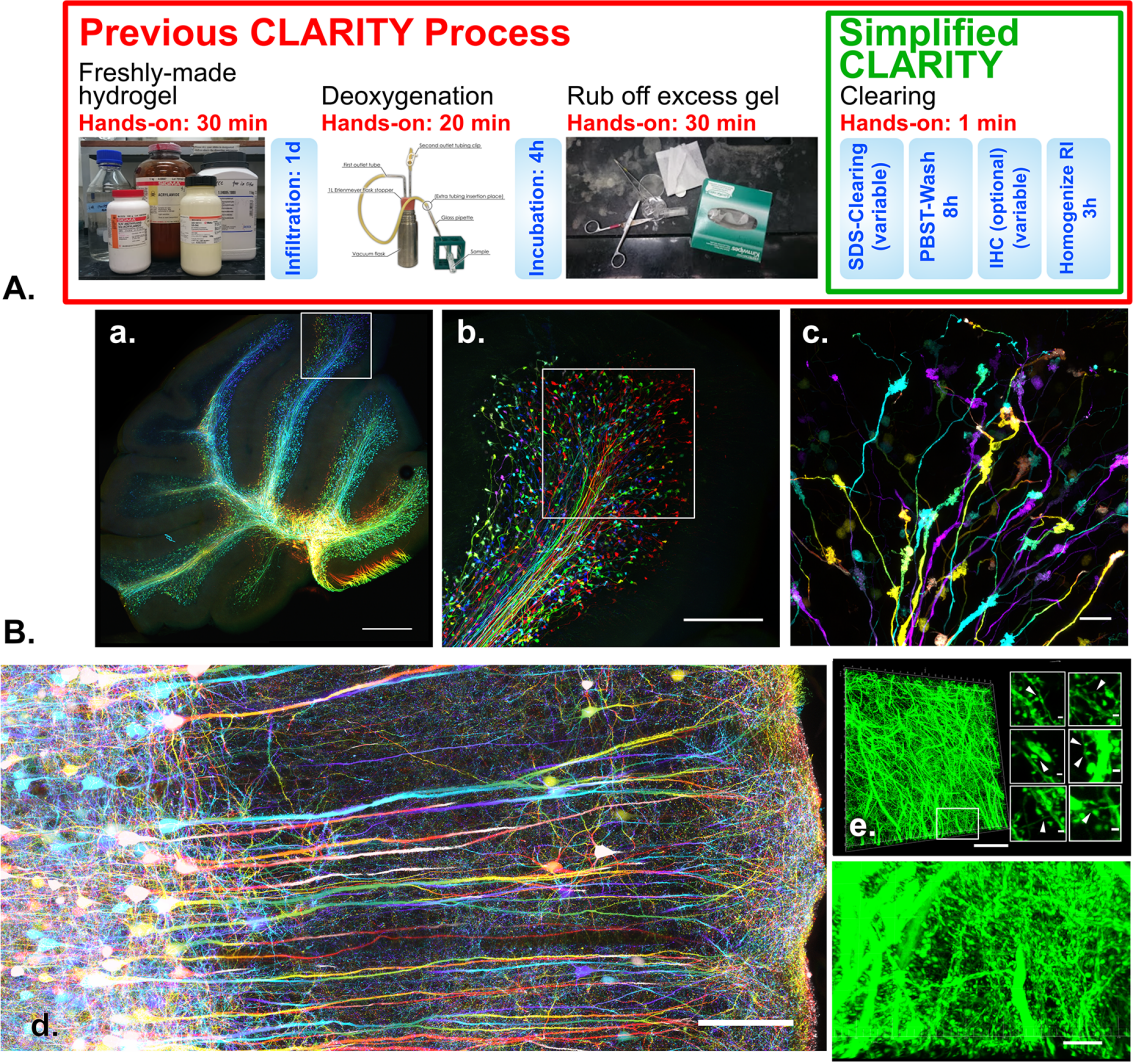
A novel, simplified CLARITY protocol proposed after this study. (**A.)** Comparison of CLARITY protocol before and after this study. The full red-boxed picture outlines the original protocol procedure, and the simplified protocol proposed after this study is outlined in the green box. The blue boxes depict the immersion/incubation steps of the tissue in different solutions. The hands-on times are indicated above each pictured procedure, which now only requires switching the tissue from one solution to another. (**B**.) Expected outcome of the simplified CLARITY method proposed in this study. All images are maximum intensity projections with color-coded Z-depths for fast rendering and better structure delineation. (**a.)** Overview of a 2 mm-thick slice of clarified Thy1-GFP transgenic mouse cerebellum with mossy fibers labelled. 4 Tiled Z-stacks (516.80 μm thick), 5x magnification. Scale bar 500 μm. (**b.)** Z-stack image (Z-depth 387.60 μm) of a folium as depicted in (**a)**, 10x magnification. Scale bar 200 μm. (**c.)** Z-stack image (Z-depth 64.69 μm) of a folium as depicted in (**b.)** under 40x magnification, Scale bar 20 μm. (**d.)** 10 tiled Z-stacks of a clarified, non-embedded mouse motor cortex, 40x magnification. Scale bar 100 μm. (**e.)** A Z-stack of dendritic fibers under 40x magnification in a dentate gyrus of a clarified Thy1-GFP mouse. Z depth 24.49 μm. Scale bar 40 μm. An enlarged view from the outlined area is depicted below the overview image. Scale bar 10 μm. The six insets to the right of the overview image show some of the numerous synapses (indicated by arrowheads) found within the entire Z-stack of fibers, with scale bars of 1 μm for reference.

To our knowledge, the current study represents the first attempt to rationalize a protocol in the field of tissue clearing, which ultimately led to our proposal of a simple, versatile method. Sample processing and staining has largely been empirical in histology since its advent in the 19^th^ century. Now, the field of three-dimensional histology is emerging thanks to modern advances in optics and novel molecular labeling methods, as well as the enabling rediscovery of tissue clearing. Our simplified approach to tissue clearing with SDS is applicable to all general laboratories without any specialized equipment or chemicals (**Fig 4A**), and should lead to the wider implementation of CLARITY for acquiring detailed biological information (**Fig 4B**). Furthermore, removal of the constraining acrylamide gel will be the prerequisite for combining expansion microscopy [3] with large tissues, which would potentially bring super-resolution images in extreme depths. We hope our work will guide and stimulate further studies on the underlying principles of other tissue clearing techniques and the development of three-dimensional histology.

## Supporting Information

S1 Fig Gross appearances of tissues cleared under different conditions. (**A.)** Human occipital cortex (1 cm thick) embedded in 0% acrylamide (i.e. non-embedded, 0%), 2% acrylamide (2%), and 4% acrylamide (4%), clarified in 4% SDS in sodium borate buffer at 55°C for 40 days. (**B.)** Electrophoretic tissue clearing (ETC) of non-embedded samples. From left to right: human hippocampus (1 mm-thick) fixed for 55 years cleared with ETC for 3 days. Mouse hemisphere fixed for 2 years cleared with ETC for 70 hours. Rat brain coronal section (1 mm) non-fixed and cleared with ETC for 70 hours. Note (1) the virtually minimal clearing seen when the fixation time in formaldehyde is long, indicating that the time course of tissue clearing depended largely on formaldehyde fixation instead of the concentration of acrylamide used for embedding; (2) the grossly deformed morphology of the non-fixed rat brain indicating that adequate fixation is essential for tissue integrity during SDS-mediated delipidation.
(TIF)

S2 Fig Analysis of amount of protein leaked per gram of tissue embedded in various formulae of acrylamide. (**A**.) Bradford assay analysis of amount of protein leaked from delipidation. 2 mm weighed slices of mouse brains (fixed for 2 days) was cleared at 37°C in 5 ml of 8% SDS in PBS after being embedded in various formulations of acrylamide/bisacrylamide. (**B**.) SDS-PAGE analysis of amount of protein leaked from 2 x 2 x 7 mm^3^ bars of human white matter (fixed for 3 weeks) for better control of tissue heterogeneity after clearing at 50°C in either 4% SDS in sodium borate buffer or 8% SDS in PBS. The gel was stained overnight in 1% Coomassie Brilliant Blue R-250, and no bands were observed in all lanes even with maximal well loading. The labels schemes were as follows: 4%, sample cleared in 4% SDS-sodium borate buffer; 8%, sample cleared in 8% SDS-PBS; the embedding formulae for each samples were depicted as (% acrylamide)/(% bisacrylamide)/(% formaldehyde) used for each sample.
(TIF)

S3 Fig Supplementary histological morphologies of non-embedded samples and acrylamide-embedded samples cleared in SDS. All imaging parameters have been controlled for each comparisons. Insets in selected figures shows enlarged views from their respective images. 4%: 4%-acrylamide embedded; NE: non-embedded samples. Scale bars 40 μm. (**A.)** Human occipital cortex stained for NF (green) and TH (red) as in **Fig 1B** with higher resolution. Z-depth 120 μm. (**B.)** Upper row: mouse spinal cord stained for ChAT, Z-depths 86.16 μm. Middle row: the same images as upper row but with thinner Z-stacks (16.16 μm) and DAPI signal rendering in order to demonstrate the anterior horn cells better. Lower row: mouse spinal cord stained for β-Tubulin III (red) with DAPI stain (blue). Z-depths 15.76 μm.
(TIF)

S4 Fig Additional surface morphologies of clarified tissues under the scanning electron microscope. Scale bar dimensions are as labelled. (**A.)** Overview of the clarified mouse cerebellum slices seen in **Fig 3** which have been embedded as labeled. A non-clarified, non-embedded control that has been processed and incubated simultaneously in PBST is provided. Note that part of the surface of the 4% acrylamide-embedded sample containing the hydrogel has been sliced off after embedding. BS: Brainstem, Cbl: Cerebellum. **(B.)** Comparison of ultrastructural morphology between the non-clarified control and a clarified sample, which has been fixed but not embedded in acrylamide. Both image series featured the granular cells of the granular layer in the mouse cerebellum. G: granular cells. (**C.)** Additional acrylamide hydrogel surface morphology seen only in the 4% acrylamide/0.05% bisacrylamide-embedded sample, either from the pure, polymerized gel itself or the surface of a tissue-hydrogel matrix. Note the pleomorphic morphology with extremely smooth surfaces unseen in natural tissues.
(TIF)

S5 Fig Original protein mass spectrometry data including non-deconvoluted data as labelled. The model reaction is provided here again for easy reference.
(TIF)

S6 Fig Collagenase-digested brain slice after acrylamide-embedding. *Clostridium histolyticum* collagenase digestion of clarified, 1% acrylamide-embedded wild-type mouse brain at 37°C, wide-field fluorescence microscopy at 5x magnification. With autofluorescence the tissue architecture is still discernible and the brain slice is largely intact. Anatomical labels: Cx: cortex, CC: corpus callosum, LV: left ventricle, IC: internal capsule, BG: basal ganglia, Fx: fornix.
(TIF)

S7 Fig Our self-assembled setup for performing Electrophoretic Tissue Clearing. Individual components are labelled in the figure.
(TIF)
