# Supplementary figures and images for: Rationalisation and Validation of an Acrylamide-Free Procedure in Three-Dimensional Histological Imaging
Source: PLoS One. 2016 Jun 30;11(6):e0158628. doi: 10.1371/journal.pone.0158628 (PMC4928791; doi:10.1371/journal.pone.0158628)

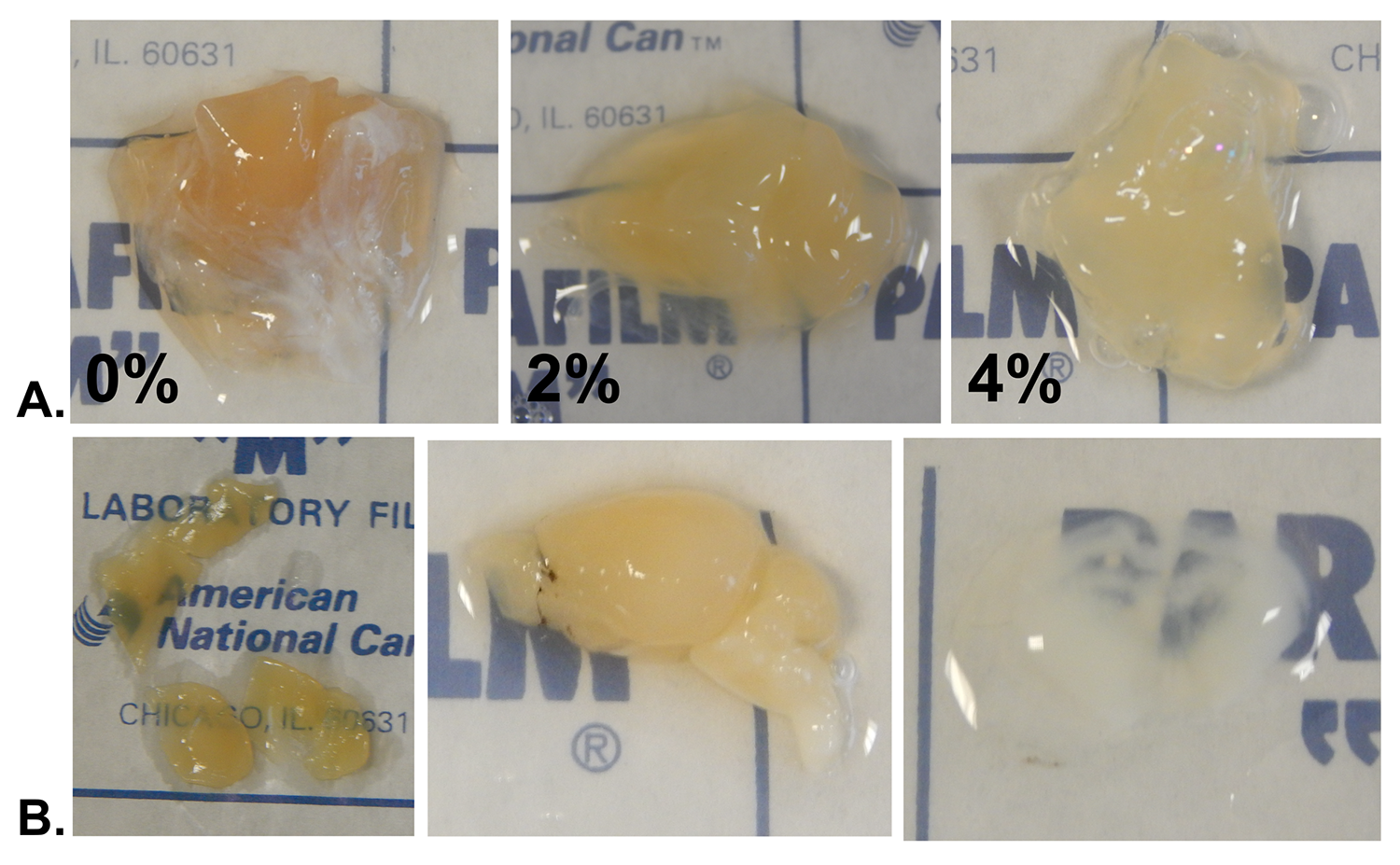

Supplement: S1 Fig — (A.) Human occipital cortex (1 cm thick) embedded in 0% acrylamide (i.e. non-embedded, 0%), 2% acrylamide (2%), and 4% acrylamide (4%), clarified in 4% SDS in sodium borate buffer at 55°C for 40 days. (B.) Electrophoretic tissue clearing (ETC) of non-embedded samples. From left to right: human hippocampus (1 mm-thick) fixed for 55 years cleared with ETC for 3 days. Mouse hemisphere fixed for 2 years cleared with ETC for 70 hours. Rat brain coronal section (1 mm) non-fixed and cleared with ETC for 70 hours. Note (1) the virtually minimal clearing seen when the fixation time in formaldehyde is long, indicating that the time course of tissue clearing depended largely on formaldehyde fixation instead of the concentration of acrylamide used for embedding; (2) the grossly deformed morphology of the non-fixed rat brain indicating that adequate fixation is essential for tissue integrity during SDS-mediated delipidation. (TIF) [file pone.0158628.s001.tif]

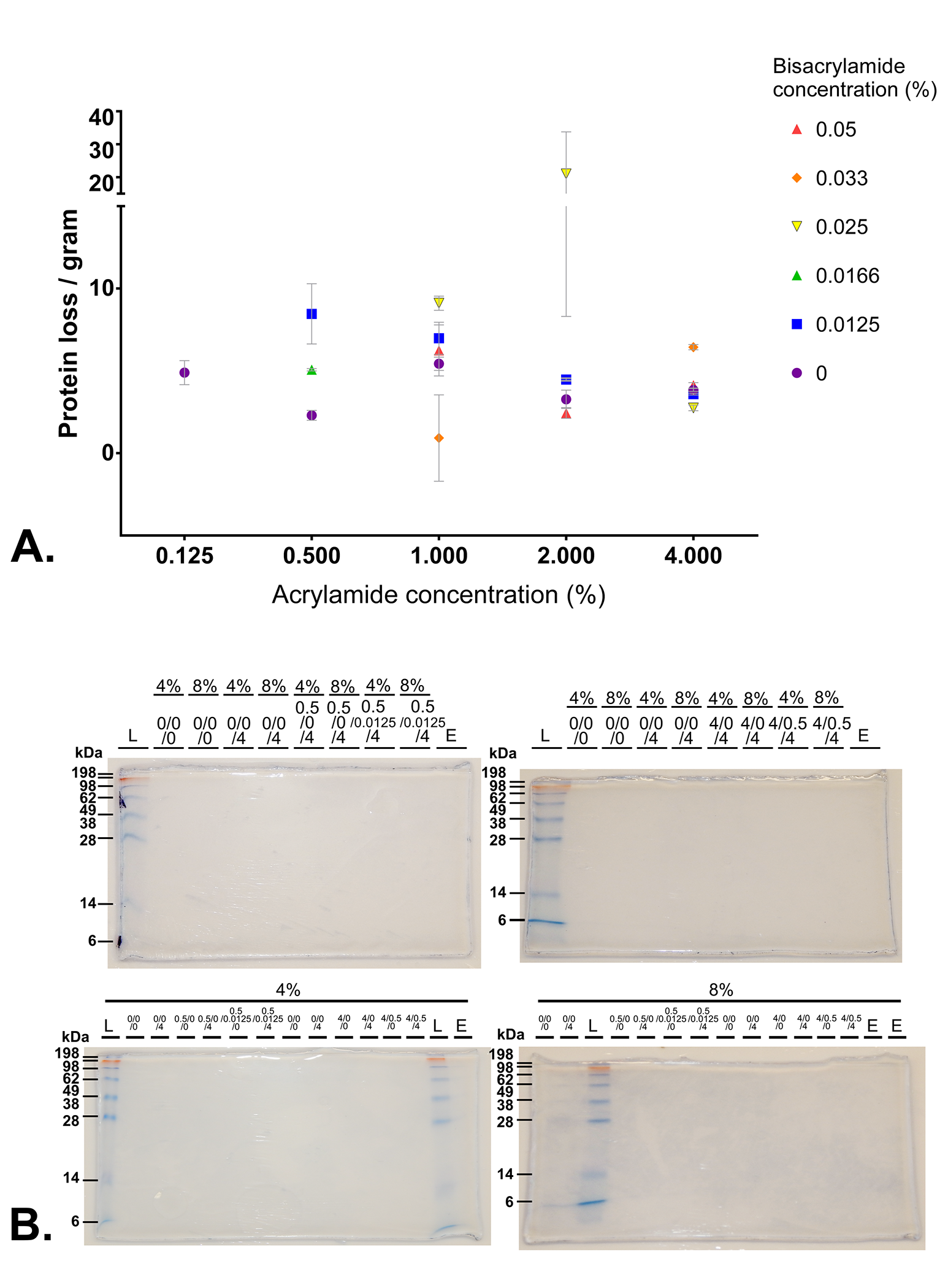

Supplement: S2 Fig — (A.) Bradford assay analysis of amount of protein leaked from delipidation. 2 mm weighed slices of mouse brains (fixed for 2 days) was cleared at 37°C in 5 ml of 8% SDS in PBS after being embedded in various formulations of acrylamide/bisacrylamide. (B.) SDS-PAGE analysis of amount of protein leaked from 2 x 2 x 7 mm3 bars of human white matter (fixed for 3 weeks) for better control of tissue heterogeneity after clearing at 50°C in either 4% SDS in sodium borate buffer or 8% SDS in PBS. The gel was stained overnight in 1% Coomassie Brilliant Blue R-250, and no bands were observed in all lanes even with maximal well loading. The labels schemes were as follows: 4%, sample cleared in 4% SDS-sodium borate buffer; 8%, sample cleared in 8% SDS-PBS; the embedding formulae for each samples were depicted as (% acrylamide)/(% bisacrylamide)/(% formaldehyde) used for each sample. (TIF) [file pone.0158628.s002.tif]

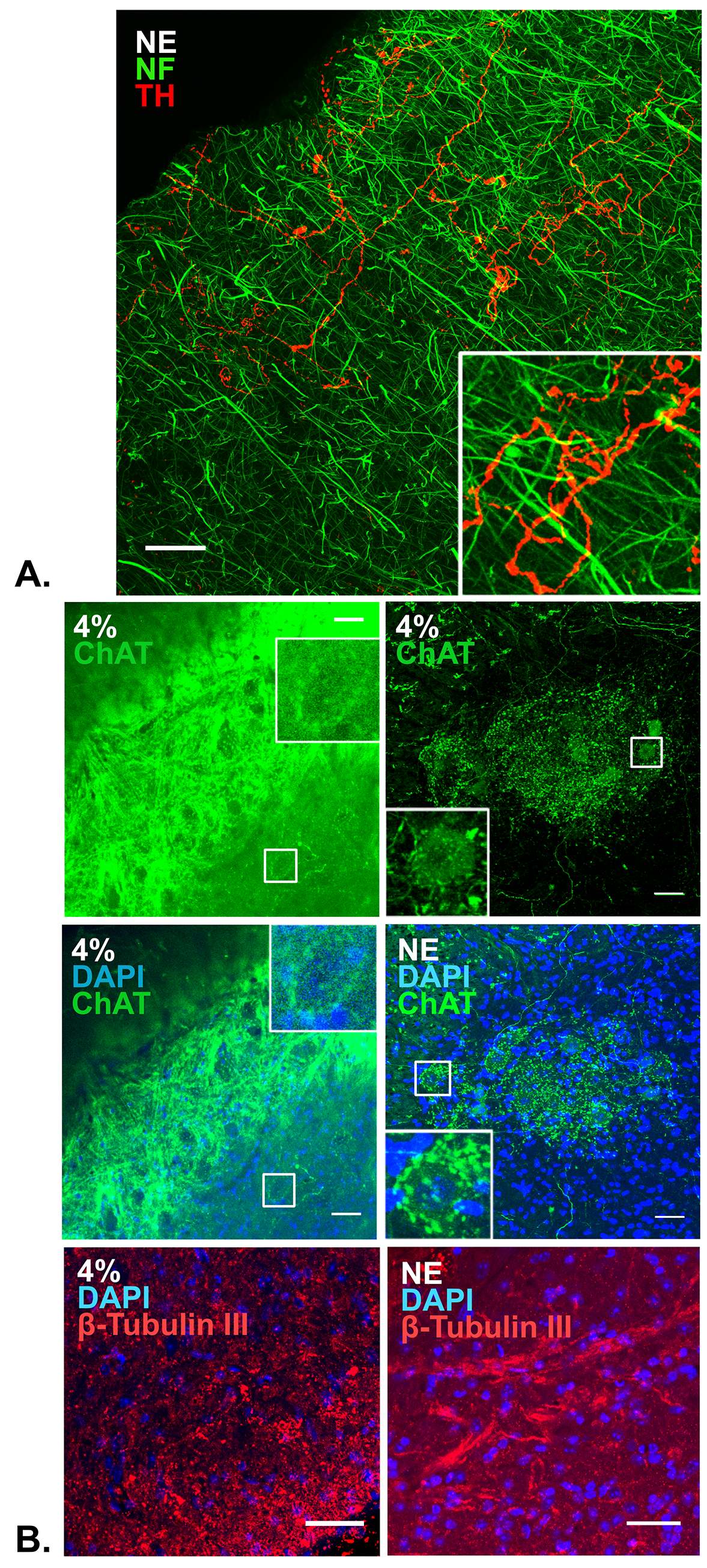

Supplement: S3 Fig — All imaging parameters have been controlled for each comparisons. Insets in selected figures shows enlarged views from their respective images. 4%: 4%-acrylamide embedded; NE: non-embedded samples. Scale bars 40 μm. (A.) Human occipital cortex stained for NF (green) and TH (red) as in Fig 1B with higher resolution. Z-depth 120 μm. (B.) Upper row: mouse spinal cord stained for ChAT, Z-depths 86.16 μm. Middle row: the same images as upper row but with thinner Z-stacks (16.16 μm) and DAPI signal rendering in order to demonstrate the anterior horn cells better. Lower row: mouse spinal cord stained for β-Tubulin III (red) with DAPI stain (blue). Z-depths 15.76 μm. (TIF) [file pone.0158628.s003.tif]

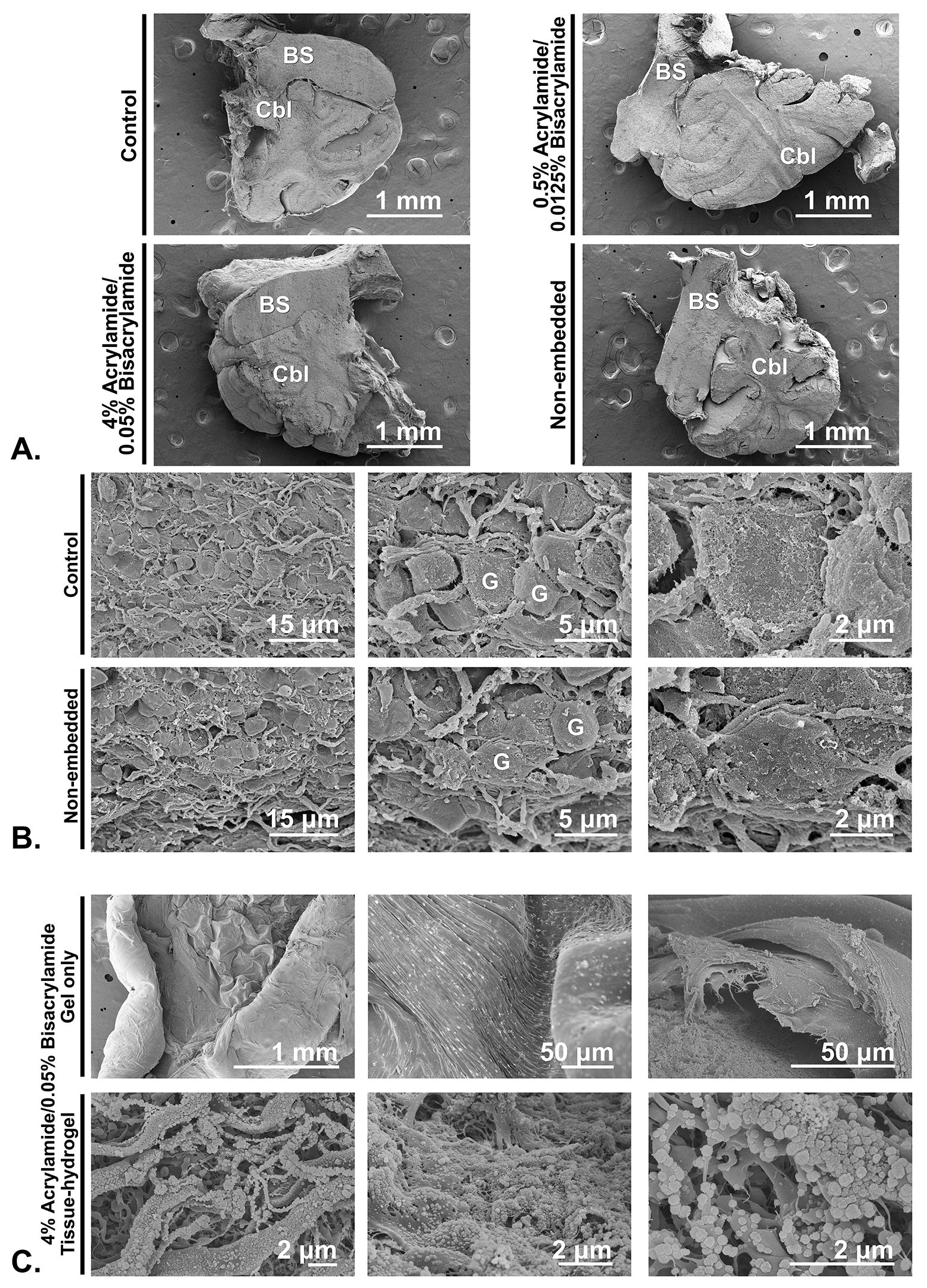

Supplement: S4 Fig — Scale bar dimensions are as labelled. (A.) Overview of the clarified mouse cerebellum slices seen in Fig 3 which have been embedded as labeled. A non-clarified, non-embedded control that has been processed and incubated simultaneously in PBST is provided. Note that part of the surface of the 4% acrylamide-embedded sample containing the hydrogel has been sliced off after embedding. BS: Brainstem, Cbl: Cerebellum. (B.) Comparison of ultrastructural morphology between the non-clarified control and a clarified sample, which has been fixed but not embedded in acrylamide. Both image series featured the granular cells of the granular layer in the mouse cerebellum. G: granular cells. (C.) Additional acrylamide hydrogel surface morphology seen only in the 4% acrylamide/0.05% bisacrylamide-embedded sample, either from the pure, polymerized gel itself or the surface of a tissue-hydrogel matrix. Note the pleomorphic morphology with extremely smooth surfaces unseen in natural tissues. (TIF) [file pone.0158628.s004.tif]

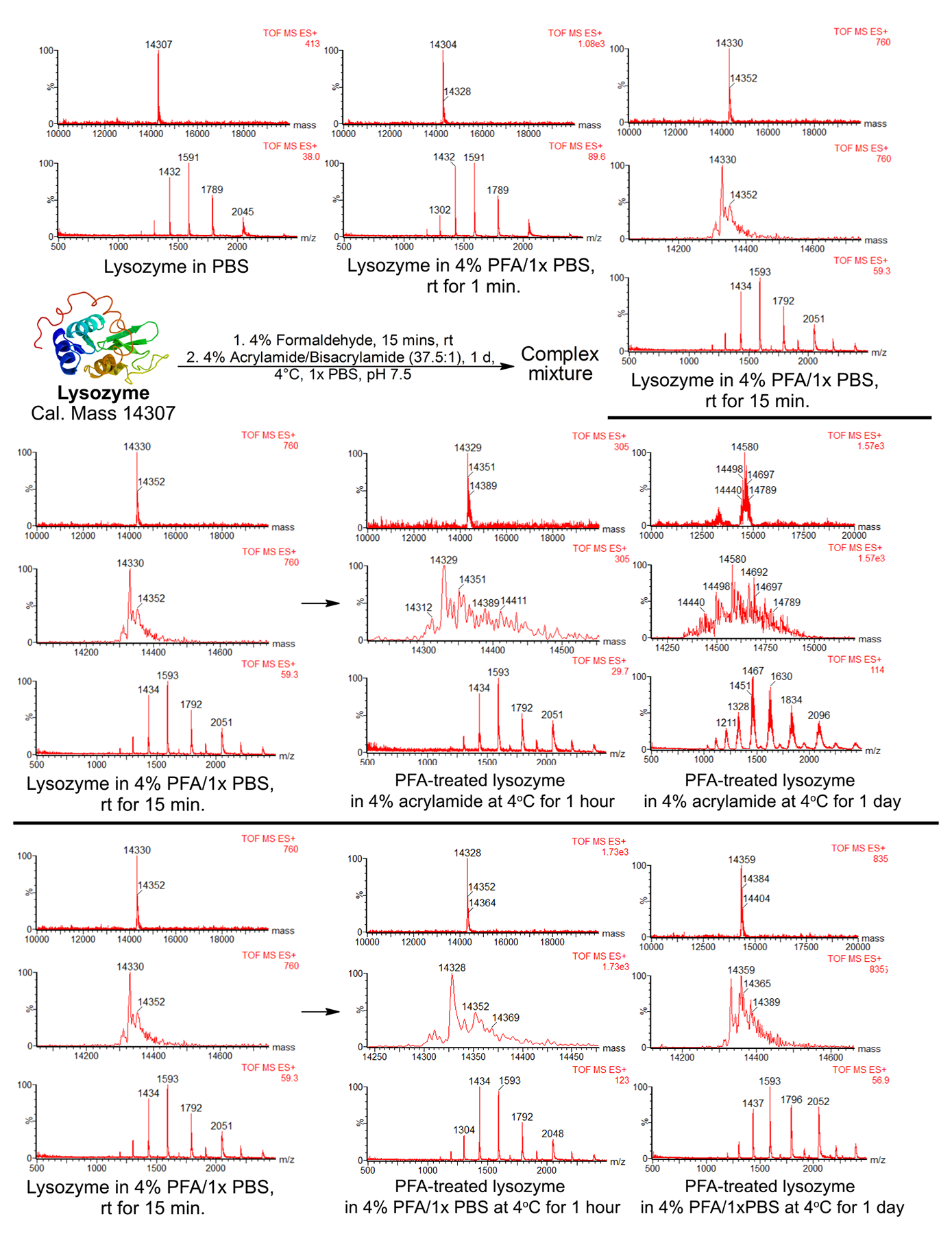

Supplement: S5 Fig — The model reaction is provided here again for easy reference. (TIF) [file pone.0158628.s005.tif]

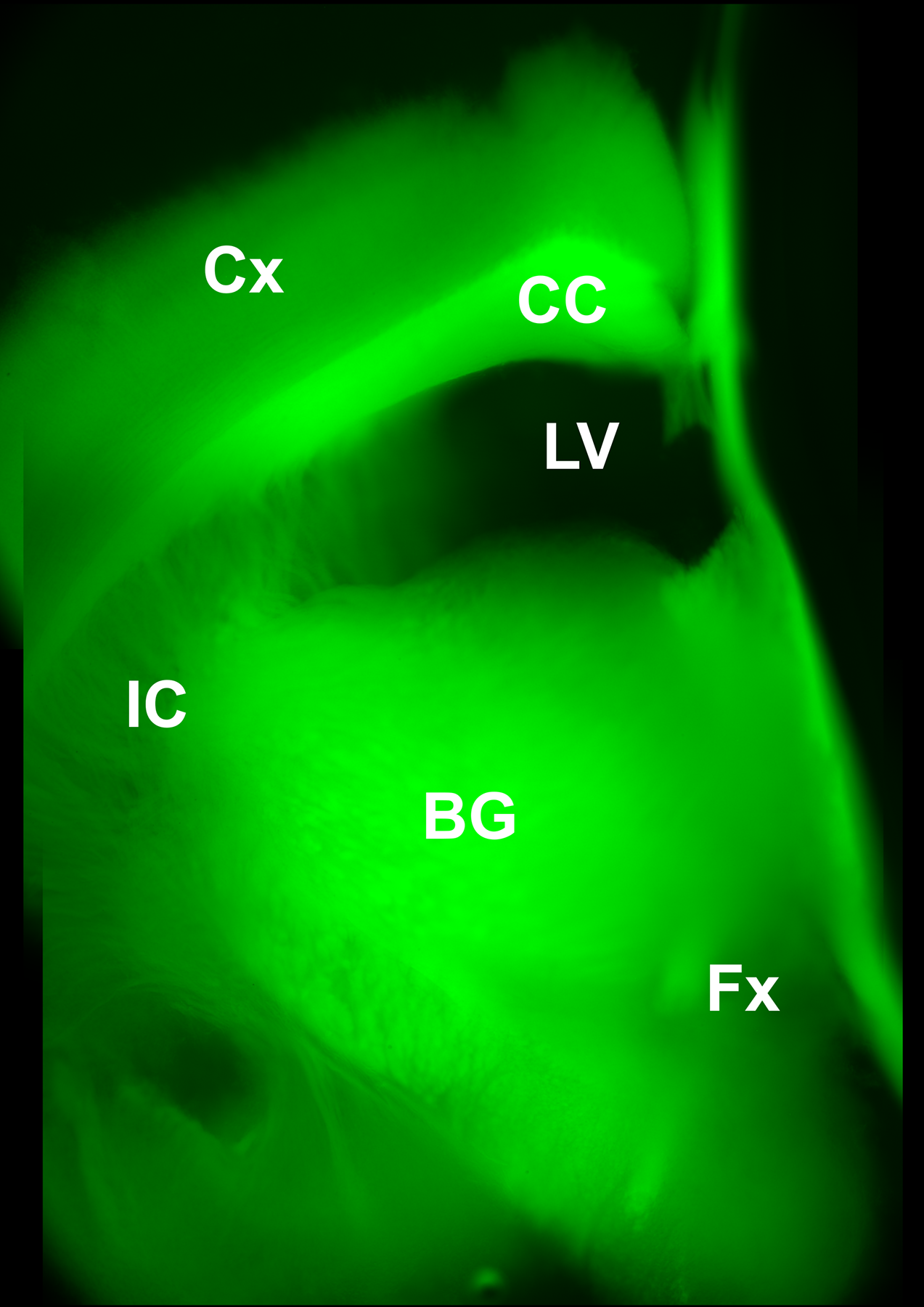

Supplement: S6 Fig — Clostridium histolyticum collagenase digestion of clarified, 1% acrylamide-embedded wild-type mouse brain at 37°C, wide-field fluorescence microscopy at 5x magnification. With autofluorescence the tissue architecture is still discernible and the brain slice is largely intact. Anatomical labels: Cx: cortex, CC: corpus callosum, LV: left ventricle, IC: internal capsule, BG: basal ganglia, Fx: fornix. (TIF) [file pone.0158628.s006.tif]

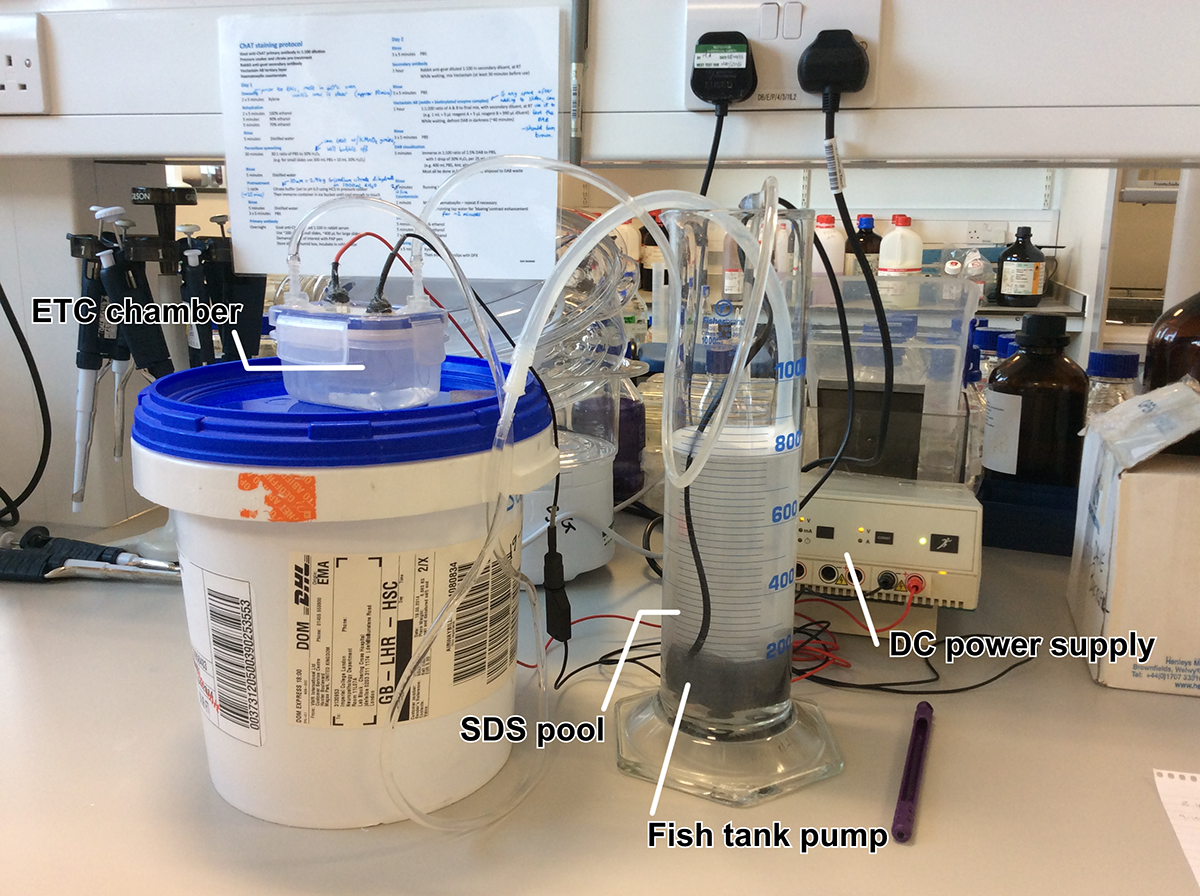

Supplement: S7 Fig — Individual components are labelled in the figure. (TIF) [file pone.0158628.s007.tif]
